# Casein phosphopeptide- amorphous calcium phosphate effects on brackets shear bond strength and enamel damage

**DOI:** 10.4317/jced.54017

**Published:** 2017-08-01

**Authors:** Roya Naseh, Farnoosh Fallahzadeh, Mohammad Atai, Omid Mortezai, Raheleh Setayeshrad

**Affiliations:** 1 Associate Professor of Orthodontics, Dental Caries Prevention Research Center, Qazvin University of Medical Sciences, Qazvin, Iran; 2 Assistant Professor of Operative Dentistry, Dental Caries Prevention Research Center, Qazvin University of Medical Sciences, Qazvin, Iran; 3 Professor of Polymer Science, Dental Caries Prevention Research Center, Qazvin University of Medical Sciences, Qazvin, Iran; 4 Assistant Professor of Orthodontics, Dental Caries Prevention Research Center, Qazvin University of Medical Sciences, Qazvin, Iran

## Abstract

**Background:**

The aim of study was to evaluate the application of casein phosphopeptide-amorphous calcium phosphate (CPP-ACP) and fluoride regarding their effect on the shear bond strength (SBS), bond failure pattern of brackets using the adhesive remnant index (ARI) and assessing the quality of enamel surface using the enamel damage index (EDI).

**Material and Methods:**

Sixty extracted premolar teeth were randomly divided into four groups regarding pretreatment application of CPP-ACP and fluoride. Brackets were bonded using the conventional method. Specimens were thermocycled for 1000 cycles and were subsequently tested for the SBS in a universal testing machine. After debonding, the teeth were examined under a stereomicroscope to evaluate the ARI. Then, The EDI was assessed using a scanning electron microscope (SEM). Data were analyzed using ANOVA and the Chi-square test.

**Results:**

Evaluation of SBS, ARI and EDI scores showed no significant difference among the study groups. However, a correlation was found between the ARI and EDI scores, indicating that with more adhesive remnants on enamel surface, enamel damage was lower.

**Conclusions:**

The use of CPP-ACP and fluoride can be considered a prophylactic application since these agents did not compromise bracket bond strength although they did not reduce iatrogenic damage to the enamel.

** Key words:**CPP-ACP, Enamel damage, SEM, Shear bond strength, ARI.

## Introduction

The enamel is the strongest part of the human body. Many studies have reported increased risk of caries creation, crack formation, and enamel decalcification during and after orthodontic treatment (1,2). Crack initiation in the enamel or damage to external tooth surface can happen as iatrogenic damage in orthodontic treatment. This damage can expose enamel prisms to the oral environment, cause decreased enamel resistance to the plaque organic acids, and facilitate enamel demineralization (3,4).

The most important way of preventing enamel demineralization is the patient’s excellent oral hygiene during orthodontic treatment (1). In addition to precise orthodontic procedures, using an agent that increases enamel strength can lead to more enamel resistance and decrease crack formation at the time of removing brackets, thereby reducing the possibility of iatrogenic damage (3). It is very important that these agents not interfere with the bracket bonding procedure and shear bond strength as this would lead to repeated bracket debonding and rebonding, which can increase the risk of enamel damage (3).

Fluoride has been shown to decrease enamel decalcification around orthodontic brackets and to increase enamel resistance to organic acids (5,6). Recently, there has been introduced a material derived from milk protein named casein phosphopeptide-amorphous calcium phosphate (CPP-ACP) to inhibit the formation of caries and induce enamel remineralization. The suggested mechanism of its action is maintaining calcium and phosphate ions in a saturation condition on tooth surface. This way, CPP-ACP can inhibit decalcification and induce remineralization (7).

Despite recommendations to use CPP-ACP in orthodontics, there is no certain information about the effects of this material on the shear bond strength of orthodontic brackets. Moreover, little studies have been done to evaluate the effect of CPP-ACP on iatrogenic enamel damage after bracket removal (8,9). Thus, the present study aims (A) to evaluate and compare CPP-ACP and fluoride in terms of their effect on the shear bond strength and bond failure pattern of brackets and (B) to assess the iatrogenic damage to the enamel after bracket removal.

## Material and Methods

This study approved by ethical committee of Qazvin University of Medical Sciences with ethical number of IR.QUMS.REC.1394.328. There is no conflict with ethical considerations. Also the study has been conducted in full accordance with the World Medical Association Declaration of Helsinki. Sixty human premolars were obtained from patients whose teeth had been extracted for orthodontic purposes and were stored in 0.1% thymol for two weeks. The samples were checked for cracks, fractures, decay, fluorosis, and other defects using a stereo microscope (MbC-2, Russia) at 20× magnification. Then, tooth surfaces were polished with pumice powder (without fluoride) to remove surface contamination and the fluoride-rich layer of the enamel. In no case was a tooth stored for more than two weeks after extraction (9).

Pretreatment and Bonding

The teeth were randomly assigned to four groups (n = 15):

• Group 1 (Control): No pretreatment of the buccal surface

• Group 2: The buccal surface was treated with a CPP-ACP paste (RecaldentTM GC Tooth Mousse, GC Europe, Leuven, Belgium).

• Group 3: The buccal surface was treated with a 0.05% sodium fluoride mouthwash (DarouTehran, Tehran, Iran).

• Group 4: The buccal surface was treated with the CPP-ACP paste and the 0.05% sodium fluoride mouthwash.

It should be noted here that in Groups 2 and 4, the CPP-ACP paste was applied to the samples for five minutes a day for one month. During the process, the teeth were placed in artificial saliva so that it could dissolve the CPP-ACP paste (10). In Group 3 and 4, the fluoride mouthwash was applied to the specimens for one minute a day for a month (10).

At the time of bonding, the specimens were initially rinsed with an air/water syringe for five seconds. Then, the enamel was trea-ted with 37% phosphoric acid (3M Dental Products, Saint Paul, MN, USA) for 30 seconds, rinsed again with the air/water syringe for 30 seconds, and dried with oil-free air for 10 seconds until a frosty-white appearance was obtained. TransbondTM XT primer (3M Unitek, Monrovia, CA, USA) was applied to the etching surface as a thin uniform coat. Stainless steel premolar brackets (G&H Orthodontics, Franklin, IN, USA) having a base area of 10.5 mm2 were bonded using TransbondTM XT composite (3M Unitek) according to the manufacturer’s instructions. Each bracket was bonded at the center of the buccal surface of the crown. After extra adhesive was removed, it was cured using a light emitting diode unit (EliparTM FreeLight 2 LED Curing Light, 3M ESPE, Saint Paul, MN, USA) for 20 seconds (10 seconds from the mesial end and 10 seconds from the distal end) (10).

Before shear bond strength was tested, samples were stored in 37˚C distilled water for 24 hours. Then, thermal cycling in deionized water was performed at temperature ranges of 5°C ± 2°C and 55°C ± 2°C for 1000 cycles. The total period of exposure to the two temperature ranges was 10 seconds, with a dwell time of five seconds in each bath in a thermocycler (Dorsa Corporation, Tehran, Iran) (11).

To test shear bond strength, the samples were placed in acrylic resin with a jig which was used to align the labial surface of each tooth so that it was perpendicular to the bottom of the acrylic mold. The samples were subsequently mounted on the lower jaw of a 4204 universal testing machine (Instron, Canton, MA, USA) so that the bracket base of each sample was parallel to the direction of the shear force. A shear force was applied to the interface between the tooth and the bracket at a crosshead speed of 1 mm per minute until bracket failure (12). The force needed to debond the bracket was recorded in newtons and converted to megapascals (MPa).

After the brackets were debonded, the adhesive remnant index (ARI) was assessed using the stereo microscope (MbC-2, Russia) at 20x magnification, and the samples were scored as follows (11):

• Score 0: No adhesive was found on tooth surface

• Score 1: Less than 50% adhesive remained on tooth surface

• Score 2: More than 50% adhesive remained on tooth surface

• Score 3: All the adhesive remained on tooth surface

Then, the remnant adhesive was removed from tooth surface using a 12-blade tungsten-carbide bur with a handpiece at a low speed, and the buccal surfaces were evaluated according to the enamel damage index (EDI) as follows (3):

• Score 0: Smooth and without crack

• Score 1: Acceptable but shallow cracks

• Score 2: Some rough cracks or shallow grooves

• Score 3: Deep and rough cracks, wide grooves, and remarkable enamel damage visible to the unarmed eye

**Figure 1:**
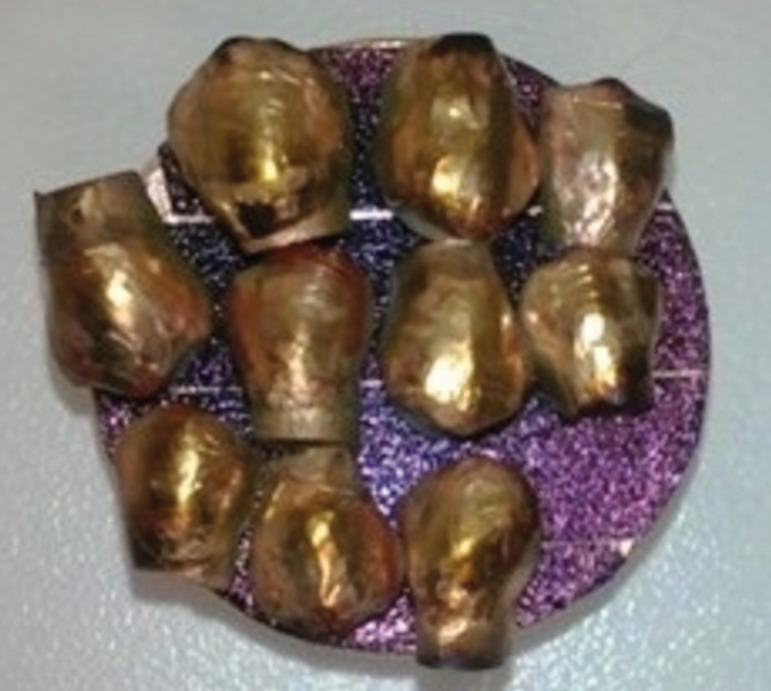
The teeth under study after being placed in the painting gold device.

**Figure 2:**
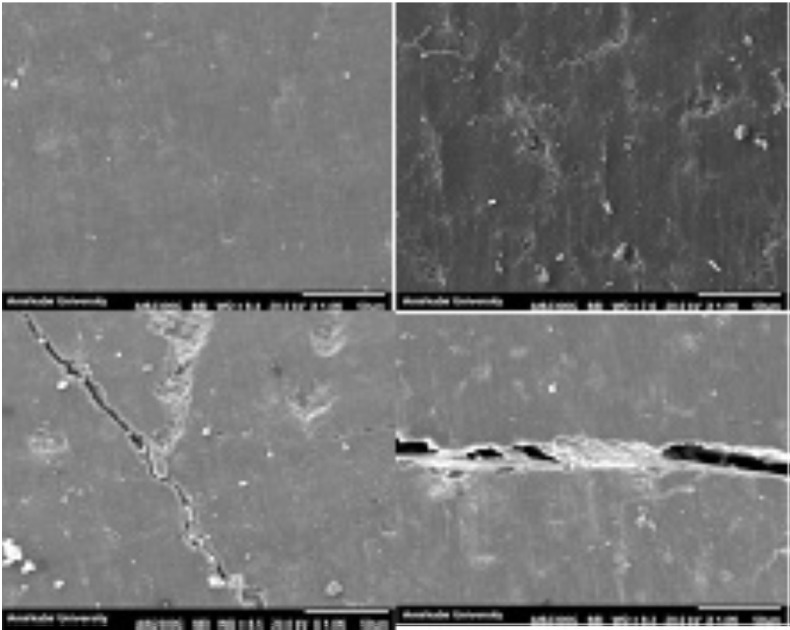
The view of enamel surface under SEM.

For EDI assessment, the samples were cut from the acrylic mold and stored in an incubator (Dorsa Corporation) at 37˚C for one week. After that, the teeth were placed in a gold painting device (DynaVac Mini Sputter Coater, Hingham, MA, USA) for 15 minutes (Fig. 1). Then, the samples were placed in a vacuum device for two hours and evaluated using a scanning electron microscope (SEM) (QuantaTM 200 FEG, FEI, Hillsboro, OR, USA) at 1000× magnification (Fig. 2) (9).

EDI and ARI assessments were performed by someone blind to the classification of the samples. Reevaluation was done for 30% of the samples by the same person three weeks later. Because the results were the same, no third evaluation was performed (13).

Once the data were gathered, they were analyzed using SPSS 21 (IBM Corporation, NY, USA, 2012).The analysis of variance (ANOVA) was used to determine whether significant differences existed among the four groups of the study regarding shear bond strength. The Chi-square test was used to determine if there were significant differences among the study groups in terms of ARI and EDI scores. A *P*-value of less than 0.05 was considered statistically significant.

## Results

The descriptive statistics for the shear bond strengths of the four study groups are presented in  Table 1.

**Table 1:**
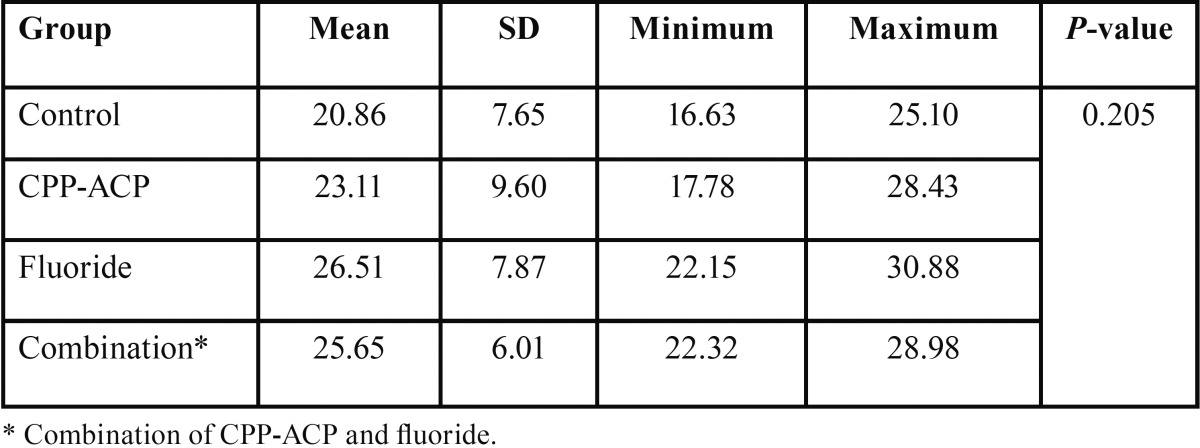
Shear bond strengths of the four study groups.

There was no significant difference among the four groups in the values of the shear bond strength (*P*=0.205).

The ARI scores for the four groups are listed in  Table 2. The Chi-square test results indicated no significant difference among the groups regarding the mode of debonding (*P*=0.054).

**Table 2:**
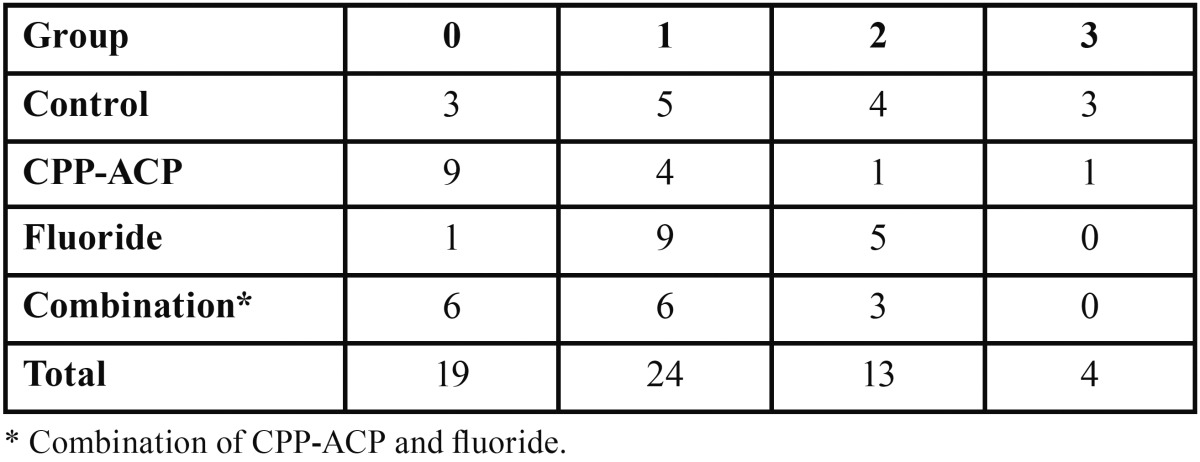
Adhesive Remnant Index (ARI) scores obtained for the study groups.

The EDI scores for the four groups of the study are given in  Table 3. The Chi-square test results showed no significant difference among the groups concerning damage to the enamel (*P*=0.153).

**Table 3:**
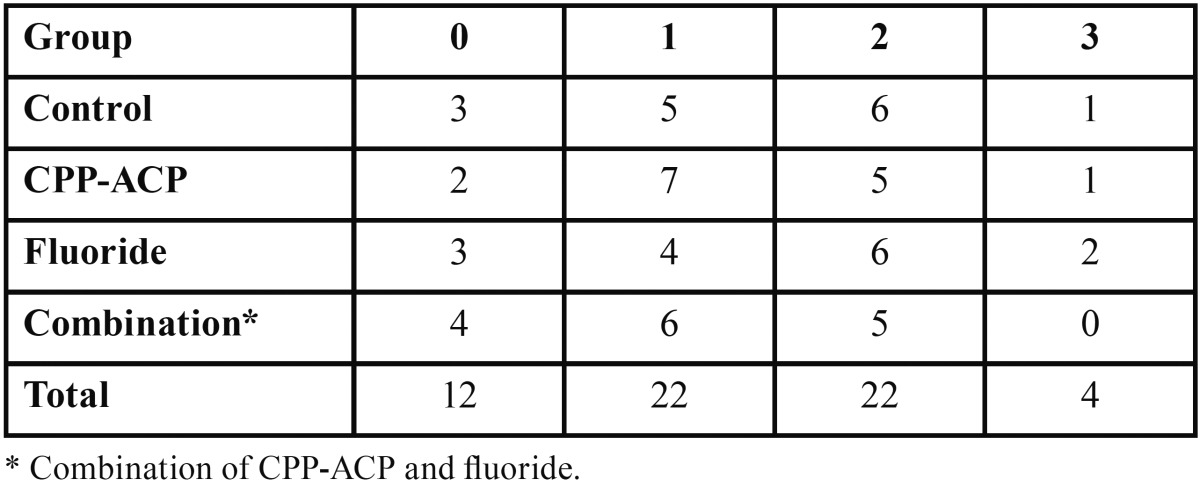
Enamel damage index (EDI) scores obtained for the four study groups.

The Chi-square analysis revealed a significant relationship between ARI and EDI scores: the lower the ARI score, the higher the EDI score (*P*=0.037).

## Discussion

The present study was an attempt to examine the effect of CPP-ACP on the shear bond strength of orthodontic brackets and to assess enamel damage at the time of bracket removal. The method of CPP-ACP application was a modification of the methods employed by Shadman (8), Bysal (6), Mayne (11), and DefneKecik (9). Shadman used a little amount of the CPP-ACP paste on enamel surface for one hour a day for five days. Bysal used the CPP-ACP solution for five minutes and then rinsed the samples with deionized water. After six hours, the topical agent (i.e., CPP-ACP) was reapplied to the tooth. Mayne used the 1% solution of CPP-ACP for one month, and DefneKecik used the CPP-ACP paste for three minutes a day. Using this paste continuously for one month is impossible in clinical use, and five minutes seems a more practical choice.

In order to simulate *in vivo* situation, when the CPP-ACP paste was applied to the teeth, artificial saliva was used to act as a sol-vent. Artificial saliva was used for two reasons: first, the producers of the CPP-ACP paste (RecaldentTM FC Tooth Mousse, GC Europe, Leuven, Belgium) recommended using saliva as a way of boosting CPP-ACP efficacy; and second, in clinical use, the CPP-ACP paste blends with saliva and using artificial saliva assimilates this environment (14). Also, 0.05% fluoride mouthwash was employed in this research because it can be easily used by patients at home on a daily basis.

In this study, the use of CPP-ACP resulted in some increase in the shear bond strength of the brackets compared with fluoride. However, this increase was not statistically significant. CPP-ACP and fluoride and the combination thereof have no adverse effect on the shear bond strength of brackets. The obtained results are similar to those of Uysal *et al.* (10) and Tabrizi *et al.* (15) In contrast, Kecik *et al.* and Xianogun *et al.* reported that CPP-ACP significantly improves shear bond strength (16,17) and Cehril *et al.* and Dunne showed that CPP-ACP reduces shear bond strength (18,19). These contradictory findings can be ascribed to the use of different durations and concentrations of CPP-ACP and fluoride.

Another finding of the present study was that fluoride mouthwash had no significant effect on shear bond strength. Some studies have speculated that fluoride could compromise the process of etching with phosphoric acid and cause weak shear bond strength (14,15,20). Smith and Gwinnet explained that interference with the formation of resin tags on enamel surface is the reason for the reduction in shear bond strength (21). Tabrizi *et al.* demonstrated that fluoride therapy reduces shear bond strength (15). However, a study by Kecik *et al.* showed an increase (16). This disparity could be due to the use of different types and concentrations of fluoride.

According to Reynolds (22), a shear bond strength of 5.9 to 7.8 MPa is sufficient for orthodontic purposes. In the present study, the application of CPP-ACP and fluoride and a combination thereof led to higher degrees of shear bond strength. Thus, the prophylactic use of these materials before bracket bonding has no adverse effect on shear bond strength.

This study also found no significant difference among the four groups in terms of ARI, but the *P*-value was 0.054, which was very close to statistical significance.

Our findings and those of others indicate that all rotary instruments that are used to remove adhesive resin remnants from tooth surfaces cause some abrasion to the enamel, which is proportional to the size and composition of the abrasive particles, the rotation speed of the instrument, and the pressure of the instrument against enamel surface (23). Thus, no instrument can achieve complete composite removal without affecting enamel surface.

Theoretically, scratches and grooves caused by the removal of resin can contribute to the formation of stains and lead to decreased resistance of the enamel to the organic acids in plaque, thus making teeth more prone to demineralization (23). However, the alterations produced on the labial surfaces of the teeth analyzed in this study were not severe enough to affect the integrity of enamel surface (23). Debonding and cleanup are operator-dependent procedures, so the results might vary from operator to operator. Since this could be a serious limitation, in our study, the same operator performed all clinical procedures.

As for the ARI, a score of 0 indicates that bond failure occurred at the adhesive-enamel interface, resulting in a greater risk of enamel damage (24). Pont *et al.* showed that the ARI is related to the decalcification of tooth surface and suggested that the scores 0, 1, and 2 could mean increased tooth surface decalcification, and application of calcium-containing agents (like CPP-ACP) would be helpful (3). In the present study, since most ARI scores of 0 were in the CPP-ACP group, we can conclude that treating the enamel using CPP-ACP before bracket bonding did not help to reduce the risk of enamel damage during bracket debonding.

Bond failure at the adhesive-bracket interface could indicate a safe debonding process because there is a faint possibility of enamel damage. Nonetheless, the removal of remaining adhesive is also important (3).

Pont *et al.* found no significant difference between ARI and EDI scores. However, in the present study, a significant difference was observed: the lower the ARI score, the higher the EDI score. This difference can be attributed to the fact that Pont *et al.* performed a microscopic evaluation of the enamel in their study.

## Conclusions

Application of CPP-ACP and fluoride before the bracket bonding procedure has no adverse effect on shear bond strength.

The use of these agents did not decrease the EDI score. Moreover, application of CPP-ACP might increase the risk of enamel damage.

Application of CPP-ACP and fluoride has no effect on the bond failure pattern and the ARI score.
